# Digging into the behaviour of an active hunting predator: arctic fox prey caching events revealed by accelerometry

**DOI:** 10.1186/s40462-021-00295-1

**Published:** 2021-11-27

**Authors:** Jeanne Clermont, Sasha Woodward-Gagné, Dominique Berteaux

**Affiliations:** 1 Canada Research Chair On Northern Biodiversity, Université du Québec À Rimouski, 300 Allée des Ursulines, Rimouski, QC G5L 3A1 Canada; 2 Center for Northern Studies, Quebec, Canada; 3 Quebec Center for Biodiversity Science, Montreal, Canada

**Keywords:** Acquisition rate, Activity budget, Behavioural classification, Biologging, Food caching, Hoarding, Predation, Predator–prey interactions, Random forest, Supervised machine learning

## Abstract

**Background:**

Biologging now allows detailed recording of animal movement, thus informing behavioural ecology in ways unthinkable just a few years ago. In particular, combining GPS and accelerometry allows spatially explicit tracking of various behaviours, including predation events in large terrestrial mammalian predators. Specifically, identification of location clusters resulting from prey handling allows efficient location of killing events. For small predators with short prey handling times, however, identifying predation events through technology remains unresolved. We propose that a promising avenue emerges when specific foraging behaviours generate diagnostic acceleration patterns. One such example is the caching behaviour of the arctic fox (*Vulpes lagopus*), an active hunting predator strongly relying on food storage when living in proximity to bird colonies.

**Methods:**

We equipped 16 Arctic foxes from Bylot Island (Nunavut, Canada) with GPS and accelerometers, yielding 23 fox-summers of movement data. Accelerometers recorded tri-axial acceleration at 50 Hz while we obtained a sample of simultaneous video recordings of fox behaviour. Multiple supervised machine learning algorithms were tested to classify accelerometry data into 4 behaviours: motionless, running, walking and digging, the latter being associated with food caching. Finally, we assessed the spatio-temporal concordance of fox digging and greater snow goose (*Anser caerulescens antlanticus*) nesting, to test the ecological relevance of our behavioural classification in a well-known study system dominated by top-down trophic interactions.

**Results:**

The random forest model yielded the best behavioural classification, with accuracies for each behaviour over 96%. Overall, arctic foxes spent 49% of the time motionless, 34% running, 9% walking, and 8% digging. The probability of digging increased with goose nest density and this result held during both goose egg incubation and brooding periods.

**Conclusions:**

Accelerometry combined with GPS allowed us to track across space and time a critical foraging behaviour from a small active hunting predator, informing on spatio-temporal distribution of predation risk in an Arctic vertebrate community. Our study opens new possibilities for assessing the foraging behaviour of terrestrial predators, a key step to disentangle the subtle mechanisms structuring many predator–prey interactions and trophic networks.

**Supplementary Information:**

The online version contains supplementary material available at 10.1186/s40462-021-00295-1.

## Background

A critical question of predator–prey dynamics is when and where do predators catch prey. However, most predators are secretive, complicating detailed assessments of their hunting strategies. Recent technology may solve this problem by revealing the behaviour of even the most cryptic species, allowing important progress in behavioural and community ecology [1–3].

With variable success, high precision GPS and accelerometers have been used to identify predation events, thus informing on the timing and location of kills as well as prey acquisition rate, a key metric to understand predator–prey relationships [4, 5]. Recently, predation events by seabirds [6, 7], fishes [8], marine [9, 10] and large terrestrial mammals [11, 12] were identified through biologging. In large terrestrial mammals, killing events of large prey can be identified through the clusters of GPS locations resulting from prey handling, which includes prey consumption and sometimes food caching [13–15]. Although this approach, which often necessitates field confirmation of kills, works for large predators, it depends on long prey handling times (and thus large prey sizes) [12, 16] matched with adequate GPS fix frequency [13, 14].

Accelerometry can inform the predator’s behavioural state and thus confirm a killing event after a cluster of GPS locations is identified [12]. Indeed, statistical tools like supervised machine learning can identify behavioural states (e.g. flying, travelling, resting, foraging) from tri-axial acceleration measurements [17, 18]. Furthermore, accelerometry may also be used to directly identify killing events of ambush predators for which killing of large prey involves stalking and high acceleration attacks [11, 19, 20]. Success of accelerometry in identifying predation events still depends on many factors like sampling regime, predator’s hunting strategy, predator and prey body sizes, and prey handling time. This explains why most accelerometry-based studies identifying predation events by terrestrial mammals are restricted to large ambush predators feeding on large prey.

Using biologging to study hunting behaviour of small active hunting predators feeding on small prey and requiring short handling times cannot rest on the identification of clustered locations. Still, studying their hunting behaviour is critical to better understand trophic networks. Detailed behavioural classification obtained from accelerometry [21, 22] may offer avenues for progress, provided the foraging behaviour of the studied species contains diagnostic acceleration patterns. For example, the fast and sharp movements of foraging razorbills (*Alca torda*) and common guillemots (*Uria aalge*) allowed researchers to quantify prey pursuit and catching through accelerometry classification [21]. Many other predators perform unique behavioural sequences potentially providing acceleration signatures of foraging events. Food caching (e.g. [23–25]) is one such sequence, as observed in many canids, which are active hunting predators storing food for later consumption [26]. Canid caching behaviour generally follows a distinctive sequence of food carrying, digging with forepaws, tamping with muzzle to press food into the soil, and head scooping to cover food with substrate [27].

We tested the potential of accelerometry to inform the hunting behaviour of an active hunting predator with short prey handling times. We did so using the arctic fox (*Vulpes lagopus*) as study model, since this small canid (ca. 2.5 kg) is a key predator over its circumpolar range, where it has been thoroughly studied [28, 29] and is well known to cache food [30, 31]. Furthermore, predation by arctic foxes generates both important top-down effects on prey populations [32] and predator-mediated interactions among prey species [33–35], thus increasing the need to understand how arctic foxes’ hunting behaviour generates a predation risk landscape [36]. Due to harsh climatic conditions and the pulsed nature of rodent populations and migratory birds in many Arctic systems, arctic foxes highly depend on food caches during periods of food scarcities such as the winter season [30, 37, 38]. On Bylot Island (Nunavut, Canada), which is home to a large greater snow goose (*Anser caerulescens antlanticus*) colony composed of > 20,000 nesting pairs [39], arctic fox summer diet is primarily composed of lemmings (*Lemmus trimucronatus* and *Dicrostonyx groenlandicus*) and goose eggs [30, 32, 39]. They can cache up to 90% of the goose eggs they collect [30]. They can also cache ca. 30% of collected goose goslings and lemmings [30]. The caching rate of eggs collected from goose nests declines from laying to hatching, but foxes recache ca. 60% of the goose eggs recovered from initial caches [30, 31]. Food caching thus represents a critical dimension of the foraging ecology of this predator. Given the stereotyped nature of food caching behaviour in canids, this behaviour could generate a spatially and temporally explicit signature of foraging events in individuals equipped with GPS and accelerometers.

Our first objective was to develop an algorithm allowing the behavioural classification of arctic fox accelerometry data and identifying prey caching events. Using in situ video calibration, we studied fox movements during two consecutive goose breeding seasons. Lemming density was low to moderate, thus most cached prey were goose eggs (see “Methods” section for details). Our second objective was to assess whether fox digging events (the most conspicuous behaviour involved in food caching) and greater snow goose nesting were spatially and temporally congruent, as a way to test the ecological relevance of our behavioural classification. We predicted that digging should occur more frequently where nest density is highest (P1), digging should occur less frequently after egg hatching (P2), as eggs become goslings that gradually disperse, and the spatial correlation between digging frequency and goose nest density should hold even after eggs have hatched (P3), since foxes recover previously cached eggs for consumption or recaching in potentially safer sites [30, 31]. Lastly, we discuss the potential to gain information on prey acquisition rates of an active hunting predator from the behavioural classification of accelerometry data.

## Methods

### Study system

We worked in May–July 2018–2019 in the southwest plain of Bylot Island (72°53′ N, 79°54′ W), in Sirmilik National Park of Canada, Nunavut. The ecosystem is characterised primarily by mesic tundra and polygonal wetlands [40]. Arctic foxes use dens to rear young and share a territory with their mating partner [41]. In 2018 and 2019, there were 115 fox dens in the study area and all were georeferenced. On Bylot, arctic foxes rely mostly on small prey, such as lemmings (40–50 g), which show important annual density fluctuations [42]. Lemming abundance was low (0.02 lemmings/km^2^) in 2018 and moderate (137 lemmings/km^2^) in 2019 as determined by capture-recapture methods [35, 43]. Foxes also collect snow goose eggs (100–150 g [37]) during the nesting period for immediate consumption or storage, as well as goslings after hatching [39]. The goose incubation period lasts 23 days from mid-June to early July, after which goose families disperse. Predation on goose nests by arctic foxes is greater when lemming abundance is low [33, 39, 44], as they then highly depend on this resource for reproduction [45]. Notably, from an isotopic analysis, Giroux et al. [45] found that geese represented up to 97% of arctic fox cubs’ diet, depending on lemming abundance and distance from the center of the goose colony. Furthermore, based on 363 h of observations inside the goose colony from June 8 to July 14 during a year of moderate lemming abundance, 75% of prey collected by foxes were goose eggs, 14% were lemmings and 11% were goslings [30].

Arctic foxes seem to cache food items individually [38], although more evidence is required on this matter regarding the smallest prey. Using radio-collared artificial eggs, Careau et al. [30] found that eggs were cached 85 m (median) from the nest. Median hoarding times (including carrying and caching times) are ca. 100 s for eggs and ca. 60 s for goslings and lemmings [30]. Foxes also opportunistically prey upon nests of other ground nesting birds such as shorebirds, passerines, and ducks, and they are their main nest predator [46, 47]. A simplified food web of the study system is available in Duchesne et al. [35].

### Fox captures, movement tracking and video observations

**Fig. 1:**
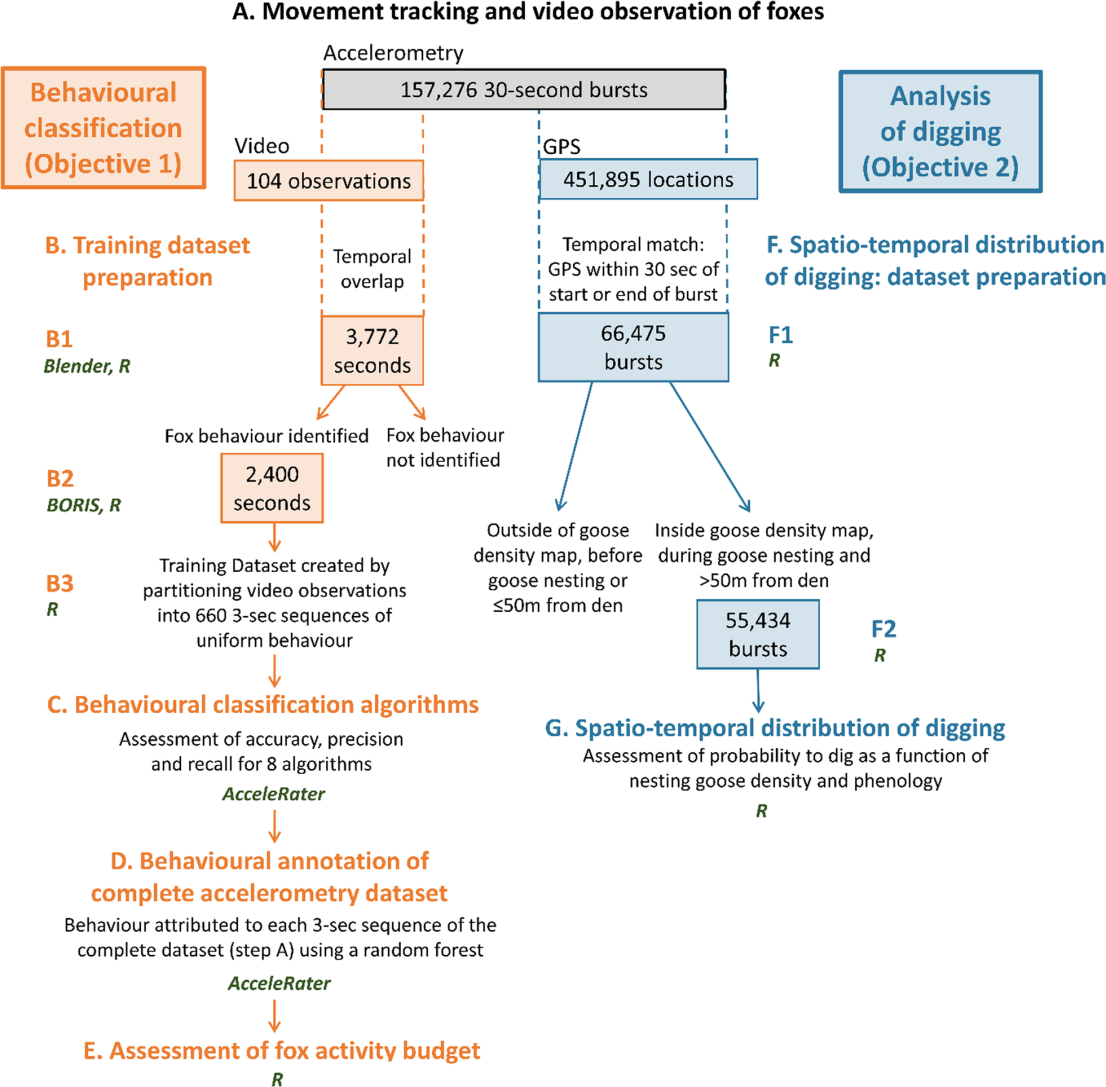
Methodological workflow for the behavioural classification of accelerometry data and assessment of activity budget (objective 1, steps **A**–**E**), and the spatio-temporal distribution of digging events (objective 2, steps **F**–**G**) in arctic fox from Bylot Island (Nunavut, Canada). The software used for data handling and analysis are indicated in dark green below each step

We captured 16 foxes using Softcatch #1 padded leghold traps (Oneida Victor Inc. Ltd., Cleveland, OH, USA), for a total of 23 fox-summers of movement data. Fox sex was determined at capture and reproductive status (yes/no) was based on whether automated cameras recorded cubs at the individual's den [48]. Each fox was marked with coloured ear tags allowing identification at a distance, and was fitted with a GPS-accelerometer collar (95 g, ca. 4% of body mass; Radio Tag-14, Milsar, Romania) equipped with rechargeable batteries, a solar panel, and UHF transmission allowing remote data download. We programmed collars to collect a GPS fix every 4 min and a 30-s accelerometry burst every 4.5 min (we unintentionally set a 4-min rather than 3.5-min break between bursts). We collected triaxial accelerometry at 50 Hz on the vertical (heave), lateral (sway) and longitudinal (surge) axes. Additional file 1: Table S1 describes sample sizes of accelerometry data for each fox and year considered in our study. After excluding data collected within two days of capture, we obtained 157,276 bursts totaling 4,718,280 s of accelerometry, and collected 451,895 GPS locations (Fig. 1A).

We videotaped collared foxes at each encounter during June and July and managed to film 15 of the 16 foxes. We collected 2.42 h of video (45 observations of 0.5–17 min) in 2018 and 6.48 h (59 observations of 1–47 min) in 2019 (Fig. 1A). We filmed a handheld GPS at the end of each video observation to allow post-synchronisation of video and accelerometry sequences.

### Objective 1: Behavioural classification from accelerometry data

#### Training dataset preparation

We first identified temporal overlaps between video and accelerometry sequences. To do so, we synchronised video times with GPS satellite time filmed at the end of the observation, using Blender video editing software (version 2.82.7 [49]). We then associated video observations to corresponding sequences of accelerometry data in R software (version 3.6.1 [50]). This yielded 3772 s of video (from 12 foxes, both years combined) that were concurrent with accelerometry data (Fig. 1B1).

**Fig. 2:**
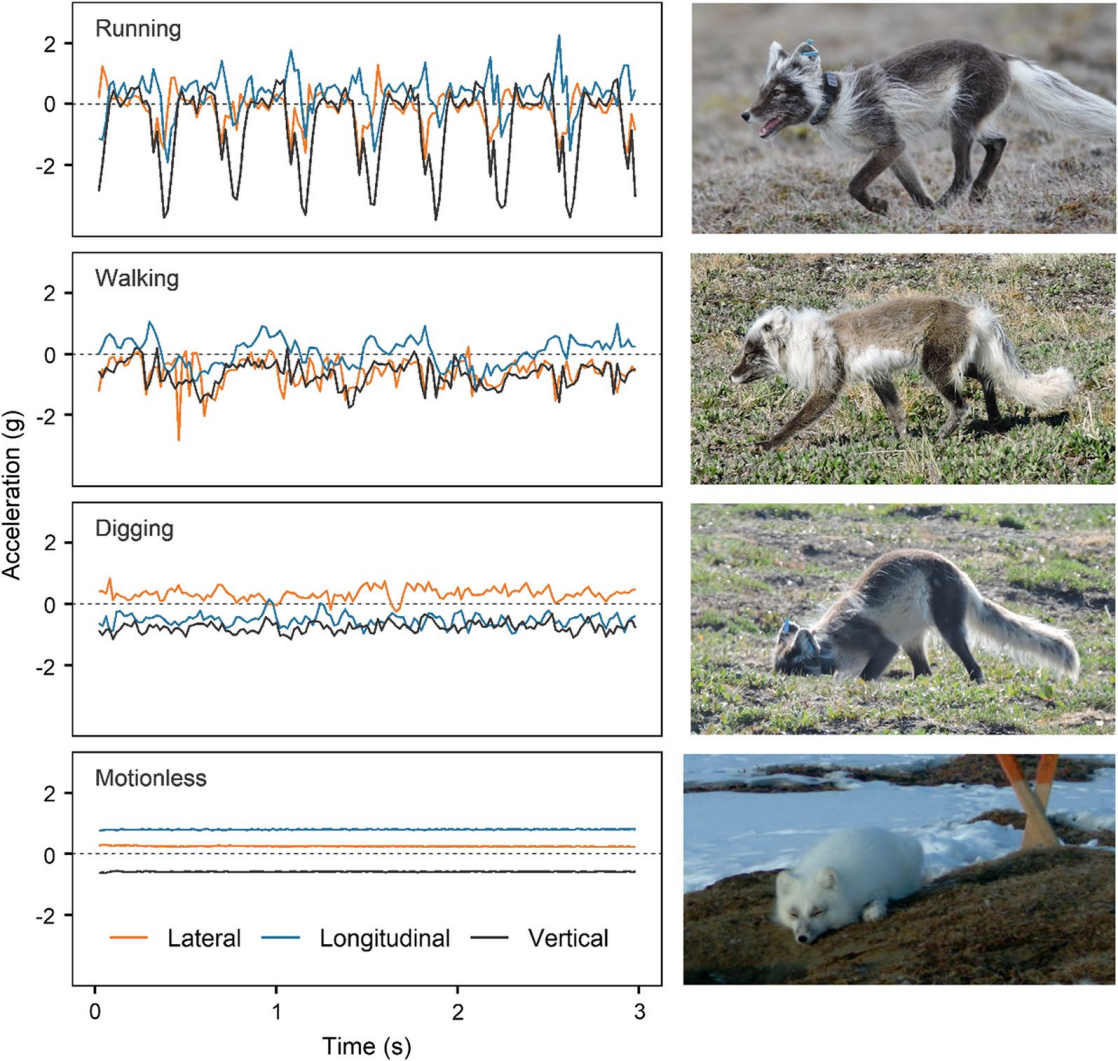
Left column: Three-second acceleration bursts on the lateral (orange), longitudinal (blue), and vertical (black) axes, for the running, walking, digging and motionless behaviour categories considered in this study. Right column: Illustration of each behaviour category. Note the various molt stages observed in these arctic foxes photographed in May–July on Bylot Island (Nunavut, Canada)

We then used BORIS software (version 7.9.7 [51]) to annotate the video observations identified above, using the detailed ethogram from Table S2 in Additional file 1. We noted the start and end times of each behaviour. Rare behaviours were ignored, and similar behaviours were grouped (Additional file 1: Table S2), yielding 4 behaviour categories: running, walking, digging and motionless (Table 1; Fig. 1). While running, walking, and motionless events are readily identified in canids [52], digging is more context-specific. In all digging events, the fox had its head close to the ground and was handling a food item (Fig. 2), mostly digging, usually tamping and scooping, and sometimes eating. The function of behavioural events grouped as digging could be identified on video observations as egg caching (44%), egg recovering (15%), or eating or recovering an unidentified food item from a ground cache (41%; Additional file 1: Table S2). Thus, during our observations, at least 59% of digging events involved a goose egg, and foxes were never seen handling a prey type other than a goose egg, suggesting that much more than 59% of digging events involved a goose egg. Movie clips are included as Additional file 2 to illustrate running, walking, digging and motionless behaviours as observed in arctic foxes from Bylot Island.

**Table 1:** Description and function of four arctic fox behaviours used for accelerometry classification, and number of 3-s sequences (N) obtained for each behaviour and used for training behavioural classification algorithms

Behaviour	Description	Function		N
Running	Fast and long-distance movement	Travel between habitat patches		146
Walking	Slow movement of short duration	Transition between running and another behaviour		126
Digging	Head down, digging, usually tamping and scooping, sometimes eating. Individual remains at a fixed location	Cache or recovery of a food item		49
Motionless	Standing, sitting or lying down (most common), with head up or down	Resting or vigilance		339

We could successfully identify fox behaviour in 2400 (63.6%) of the 3772 s of video observations that were concurrent with accelerometry data (Fig. 1B2). We prepared our training dataset by partitioning these data into 3-s sequences that each contained a single, uninterrupted behaviour (thus excluding brief sequences of behaviours such as standing between two running sequences). This yielded 660 sequences representing 1980 s of accelerometry (Table 1; Fig. 1B3). A training dataset composed of 5-s sequences yielded similar model accuracies but lower precisions, so we used sequences of 3 s to increase observation sample size and precision. Figure 2 presents an example of a 3-s sequence of acceleration for each behaviour.

#### Behavioural classification algorithms

We used the open-access web application AcceleRater [53] to train behavioural classification algorithms based on our training dataset (Fig. 1C). We computed 52 summary statistics [53] for each of the 660 3-s sequences of the training dataset. Averages and standard deviations of the 52 summary statistics obtained for training sequences are presented for each behaviour category in Additional file 1: Table S3, and among-behaviour differences in the summary statistics are also illustrated in Additional file 1: Figs. A–R.

We trained 8 algorithms, including a three nearest neighbours algorithm, a linear support-vector machine, a radial basis function kernel SVM, a decision tree, a random forest, a gaussian naïve Bayes, a linear discriminant analysis and an artificial neural network [17, 53, 54]. We used a five-fold cross-validation method to assess training accuracy, precision and recall for each behaviour. This method splits the dataset into 5 equal parts containing 20% of the dataset, uses 4 parts for training and the remaining one for validation. The cross-validation was repeated 5 times, with each part used once for validation.

To identify which algorithm performed the best at classifying our data, a confusion matrix was built in AcceleRater for each algorithm to represent correct and incorrect classifications, and count true positives (TP), true negatives (TN), false positives (FP), and false negatives (FN). Accuracy, precision and recall were calculated for each behaviour category to assess classification performance. Accuracy is the proportion of correct classifications either into or out of a given behaviour category:

$$accuracy = \frac{TP + TN}{{TP + TN + FP + FN}}$$

Precision is the proportion of classifications into a given behaviour category that were correct. Higher precision indicates fewer false positives:

$$precision = \frac{TP}{{TP + FP}}$$

Recall is the proportion of instances of a behaviour classified into the correct category. Higher recall indicates fewer false negatives:

$$recall = \frac{TP}{{TP + FN}}$$

Lastly, we used AcceleRater to annotate our complete accelerometry dataset (157,276 30-s bursts) previously partitioned into 3-s sequences in R (Fig. 1D). We used the random forest algorithm which showed the greatest performance for all metrics (see “Results” section). We then determined fox activity budgets by calculating proportions of the dataset associated to each behaviour (Fig. 1E).

### Objective 2: Spatio-temporal distribution of digging behaviour

#### Data preparation

To assess the spatio-temporal distribution of digging in relation to nesting goose density and phenology, each 30-s accelerometry burst of the complete dataset (157,276 bursts) was associated to the closest GPS location, provided the time stamp of the GPS location was within 30 s of the start or end of the burst, which was the case for 42.3% (66,475) of the bursts (Fig. 1F1). We then associated to each burst location the local nesting goose density (individual geese/ha), a proxy for nest density that was estimated from detailed field surveys performed in 2018 and 2019 [40]. We also determined whether each burst occurred during the goose incubation or brooding (when goslings disperse) period, based on starting and ending dates of incubation and brooding for each year (incubation start dates: June 19 in 2018, June 12 in 2019; brooding start dates: July 12 in 2018, July 5 in 2019; brooding end dates: August 3 in 2018, July 27 in 2019), as provided in Grenier-Potvin et al. [40]. In addition, we calculated for each burst the distance to the nearest den (m). We then excluded from analyses 11,041 bursts (Fig. 1F2) that (1) were located outside of the snow goose density map (5563 bursts), (2) were collected before the beginning of the goose nesting period (2359 bursts; no bursts were collected after goose nesting), or (3) occurred within 50 m of a den, as digging may then be associated with den maintenance rather than foraging (3119 bursts). This data preparation thus allowed us to assess whether digging events occurred during each of 55,434 30-s bursts (35.2% of the complete dataset).

#### Statistical analysis

We used a generalised linear mixed model (R package lme4 [55]) with a binomial distribution and a cloglog-link function to predict the probability that a fox engaged in digging during a 30-s acceleration burst (0 = no digging event, 1 =  ≥ 1 digging event), with respect to nesting goose density (P1), goose reproduction period (P2) and their interaction (P3), all included as fixed effects (Fig. 1G). We also included sex, reproductive status, and their interaction as fixed effects, as these factors may affect fox behaviour and thus represent confounding variables. Fox ID and year were fitted as random effects. Nesting goose density was centered and standardised to facilitate interpretation of model estimates [56]. We used as reference values in the model period = incubation, sex = male, and reproductive status = reproductive.

## Results

### Behavioural classification of accelerometry data

The random forest model yielded the greatest average accuracy, precision and recall values compared to other algorithms, and it provided a good classification of the 4 behaviours, with accuracies > 96% (Table 2). Most importantly, it yielded by far the greatest precision for digging (92.5%, compared values are identified with an asterisk in Table 2) and thus the fewest number of false positives for this behaviour, which was required to address our second objective. The random forest however yielded a recall value that was lower for digging (75.5%, Table 2) than for the other behaviours, due to a greater proportion of digging false negatives (12 out of the 49 sequences of digging were false negatives, Table 3). Digging false negatives generated a small proportion of false positives in other behaviour categories, which were much more frequent in the data (Table 3). As a result, all behaviours were classified by the random forest with a precision > 90%.

**Table 2:** Accuracy, precision and recall values obtained for the 4 behaviour categories, for each algorithm. The weighted average across behaviour categories is also given

Algorithm	Classification performance	Running	Walking	Digging	Motionless	Weighted average
Three nearest neighbours	Accuracy	98.18	96.82	97.12	97.58	97.53
	Precision	95.27	91.34	*80.00	98.21	94.90
	Recall	96.58	92.06	81.63	97.05	94.85
Linear support-vector machine	Accuracy	96.36	95.30	94.39	96.67	96.17
	Precision	93.57	87.40	*60.00	97.60	91.97
	Recall	89.73	88.10	73.47	95.87	91.36
Radial basis function kernel SVM	Accuracy	97.12	96.82	96.67	96.97	96.95
	Precision	93.79	92.68	*75.47	97.05	93.89
	Recall	93.15	90.48	81.63	97.05	93.79
Decision tree	Accuracy	97.27	96.21	95.15	97.42	96.99
	Precision	93.84	90.40	*64.41	98.79	93.54
	Recall	93.84	89.68	77.55	96.17	93.03
**Random forest**	**Accuracy**	**97.58**	**96.67**	**97.73**	**98.03**	**97.65**
	**Precision**	**92.76**	**90.63**	*92.50	**97.94**	**95.00**
	**Recall**	**96.58**	**92.06**	**75.51**	**98.23**	**95.00**
Gaussian Naïve Bayes	Accuracy	97.73	96.82	95.61	97.73	97.40
	Precision	93.38	98.17	*65.15	98.50	94.83
	Recall	96.58	84.92	87.76	97.05	93.94
Linear discriminant analysis	Accuracy	98.33	95.91	95.45	97.88	97.42
	Precision	97.20	88.37	*67.27	98.80	94.11
	Recall	95.21	90.48	75.51	97.05	93.79
Artificial neural network	Accuracy	97.42	96.52	96.82	97.42	97.21
	Precision	93.88	89.92	*81.82	97.35	94.01
	Recall	94.52	92.06	73.47	97.64	94.09

Asterisks allow easy comparison of precision across algorithms for digging. The random forest model was retained and is in bold

**Table 3:** Random forest confusion matrix with actual and predicted number of observations for each behaviour category

	Predicted category				Total
	Running	Walking	Digging	Motionless	
**Actual category**					
**Running**	141	4	1	0	146
**Walking**	5	116	2	3	126
**Digging**	3	5	37	4	49
**Motionless**	3	3	0	333	339
**Total**	152	128	40	340	660

Thus, we retained the random forest model to annotate our complete accelerometry dataset. Only 7.5% of the 3-s sequences classified as digging by this algorithm were done so wrongly, while the random forest missed 24.5% of digging sequences. The random forest was therefore conservative when assigning digging to a given sequence.

### Arctic fox activity budget

Classification of our complete accelerometry dataset (Fig. 1E) confirmed that arctic foxes are active hunting predators, as 50.7% of their activity budget was devoted to active behaviours, specifically running (34.0%), walking (8.5%), and digging (8.2%). This left 49.3% of their activity budget devoted to motionless behaviours. These proportions may however be very slightly overestimated, as running, walking, digging and motionless composed ca. 97% of fox behaviours in the video observations used to create our training dataset (Additional file 1: Table S2).

### Spatio-temporal distribution of digging behaviour

**Fig. 3:**
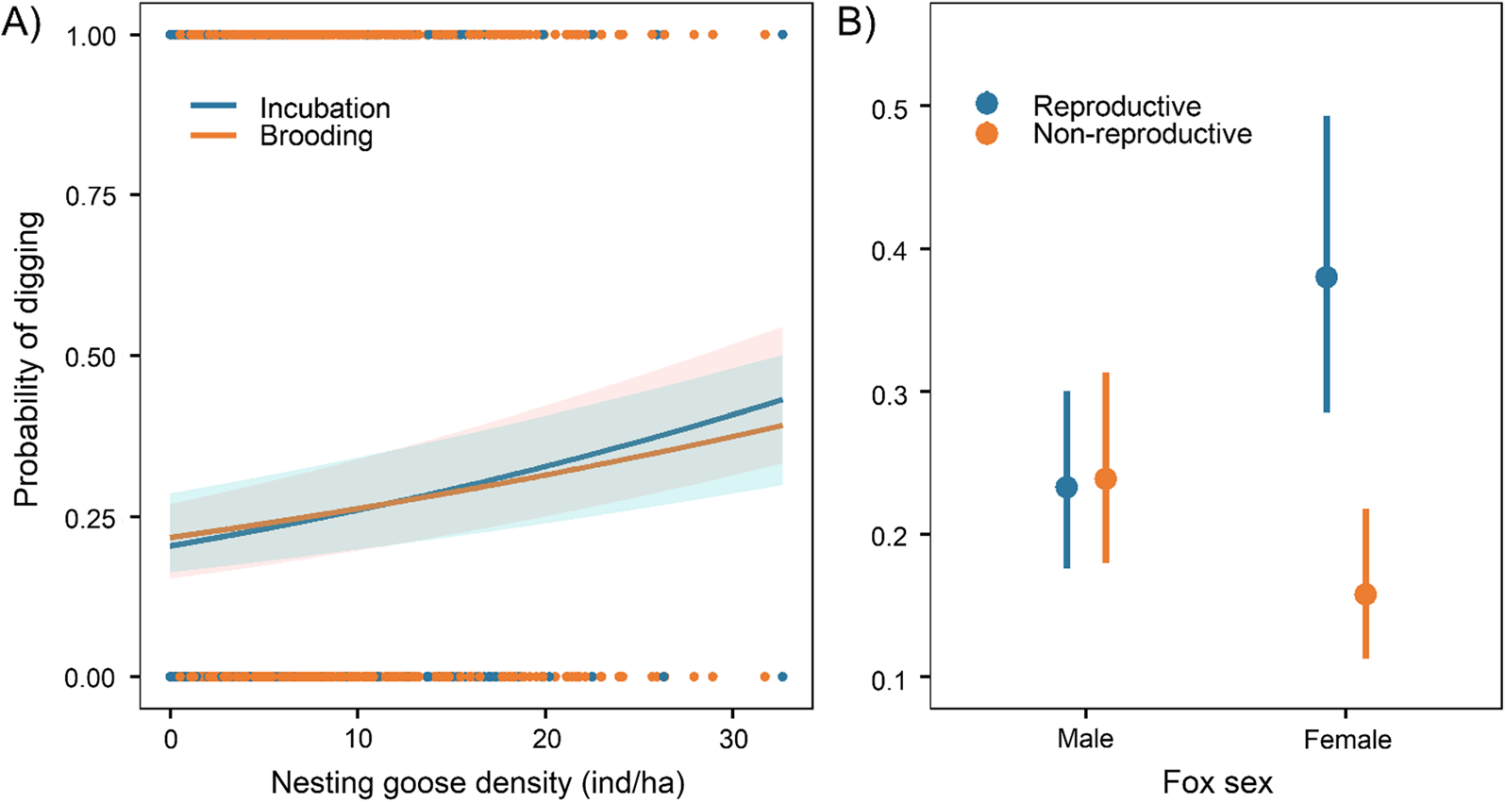
Predicted probability of digging during a 30-s acceleration burst (burst interval 4.5 min) as a function of **A** nesting goose density (a proxy for goose nest density) and goose reproduction period (incubation in blue, brooding in orange), and **B** sex and reproductive status (reproductive in blue, non-reproductive in orange). Nesting goose density was centered and standardised in the model, then back-transformed before plotting. Model reference values are fox sex = male, fox reproductive status = reproductive, period = incubation and nesting goose density = average

Foxes engaged in digging in 31.1% of the 55,434 30-s bursts retained for analysis of the spatio-temporal distribution of digging (Fig. 1G), justifying the use of the cloglog-link function in our binomial model since the ratio of 0:1 values was 69:31. Probability of digging increased with nesting goose density (P1 supported) and was slightly lower during brooding compared to incubation when nesting goose density was > ca. 12 ind/ha (P2 partly supported; Table 4; Fig. 3A). The effect of nesting goose density on the probability of digging was consistent across goose reproduction periods (P3 supported; Table 4; Fig. 3A). Probability of digging was the highest for reproductive females and the lowest for non-reproductive females, compared to reproductive and non-reproductive males who showed intermediate values (Table 4; Fig. 3B).

**Table 4:** Results of the generalised linear mixed model (on the cloglog-scale) explaining the probability of engaging in digging behaviour (binomial distribution), as a function of nesting goose density, goose reproduction period (incubation versus brooding), interaction between goose density and reproduction period, as well as fox sex, reproductive status and their interaction (n = 55,434 30-s bursts of accelerometry collected on 23 fox-years)

Fixed effect	Estimate [95% CI]	SE	*z*-value	*p*-value
Intercept	− 1.35 [− 1.68, − 1.01]	0.16	− 8.31	< 0.001
Nesting goose density	0.16 [0.14, 0.18]	0.01	15.54	< 0.001
Goose reproduction period	0.04 [0.01, 0.07]	0.02	2.50	0.01
Density: period	− 0.03 [− 0.06, − 0.01]	0.01	− 2.38	0.02
Fox sex	0.59 [0.10, 1.07]	0.23	2.56	0.01
Fox reproductive status	0.03 [− 0.07, 0.12]	0.05	0.55	0.59
Sex: status	− 1.05 [− 1.16, − 0.94]	0.06	− 18.05	< 0.001

We used as reference values nesting goose density = average, period = incubation, sex = male, and reproductive status = reproductive. Nesting goose density, a proxy for goose nest density, was centered and standardised in the model. Variance and standard error were 0.21 and 0.46 for fox ID and 0.005 and 0.07 for year

## Discussion

We demonstrated that high precision movement data can indirectly inform hunting behaviour of a terrestrial, active hunting predator feeding on small prey requiring short handling times. Through the behavioural classification of accelerometry data, we detected events of digging, a behaviour that our detailed field observations associated with food caching. Our methodology should be applicable to other small carnivore species that cache their food, such as canids and felids [26], or more broadly to any predator using foraging behaviours resulting in diagnostic acceleration patterns. Furthermore, accelerometry combined with geolocation indicated when and where arctic foxes cached their prey, here goose eggs. This proved to have strong ecological relevance, since the spatial and temporal availability of goose eggs shaped the probability of digging. These findings open new opportunities for discovery. Most notably, predator hunting generates spatial variation in predation risk, which shapes prey behaviour and ultimately the structure of prey communities [36, 57]. Therefore, detailed assessments of predator activity budgets and identification of key hunting behaviours are important steps to decipher the mechanisms driving local biodiversity, at least in ecosystems strongly driven by top-down trophic interactions.

### Accelerometry reveals prey caching events

Using a random forest, we classified accelerometry data into the four main behaviours composing ca. 97% (according to our training dataset) of an arctic fox activity budget: running, walking, digging and motionless. Our classification was 98% accurate, well in line with the performance reached with other predator species [3, 11, 58] although directly comparing studies is risky due to varying methods and studied behaviours. Our field observations show that digging behaviour can be mostly attributed to snow goose egg caching in our study system, and we know that foxes forage selectively in patches of high nesting goose density [40]. In good agreement with the above, we found that the probability of digging increased with nesting goose density. However, the strength of the relationship between the probability to dig and nesting goose density may have been decreased by habitat characteristics and specificities of fox caching behaviour. First, patches of high goose nest density tend to occur in the most complex habitats, like polygonal wetlands [59], in which fox attacks on goose nests are generally less successful, thus reducing egg acquisition and caching rates [31, 44]. Second, foxes cache eggs 85 m away (median) from predated nests [30] and goose nest density is rather patchy at this scale. Thus, nest density may differ between sites of egg collection and egg caching. Further research should seek to refine our understanding of the spatial distribution of fox digging in the greater snow goose colony of Bylot Island. More generally, our study should be considered as a first step in the use of accelerometry to model foraging behaviour of a small terrestrial carnivore.

We also found that the probability of digging by foxes was mostly similar between the goose incubation and brooding periods. This is counterintuitive since egg availability should obviously decrease after hatching. Yet, in our study system, the rate of egg recovery and recaching was shown to increase over the incubation period as foxes manage their stored food [31]. We could expect recovery rate to continue to increase after hatching, when food availability drops, thus reconciling apparently conflicting evidence. Moreover, our results show that after egg hatching, recoveries for consumption or recaching were more likely to occur in areas where goose nest density was highest, that is where a greater proportion of caches were initially made during the incubation period. Our results provide new insights on arctic fox foraging behaviour, but a finer classification of accelerometry data, with more detailed behaviours labeled, would strongly enlighten the complex dynamic of prey acquisition, caching, recovery, recaching, consumption, and even pilfering, in a predator–prey system characterized by pulsed resources, food storage, and delayed food consumption.

### Prey caching events inform arctic fox foraging and predator–prey interactions

Our analysis of potentially confounding variables on the probability of digging suggested that reproductive females were more likely to dig (and thus perform egg caching or recovery) than males and non-reproductive females. If confirmed by larger sample sizes, such variation in the frequency of digging across sex and reproductive classes opens the door to productive tests of hypotheses. For example, more food caching by reproductive females than by males might indicate greater parental investment. Alternatively, reproductive males may prefer to bring food to the den to feed the female and the young, instead of caching it. Testing these hypotheses using accelerometry could quickly enhance our understanding of reproductive and movement ecology in arctic foxes and many other small to medium size predators.

Furthermore, as arctic foxes are territorial and tend to avoid territory borders [40], their territoriality could lead to non-random distribution of specific behaviours. For example, caches could be preferentially located away from territory edges to reduce pilferage, as observed in wolverines (*Gulo gulo*) that tend to cache food in sites less exposed to competitors [25]. Another interesting avenue would be to directly assess arctic fox tendencies to do cache pilfering in neighbour territories or along overlapping areas. Interestingly, Samelius and Alisaukas [38] observed on Banks Island (Canada) that during years where arctic foxes were very abundant, they recovered and moved cached eggs at a higher rate, potentially due to increased cache pilfering.

In our study area, foxes select habitats where lemmings and geese are most abundant [40]. This generates spatial variation in predation risk, with cascading effects on nest site selection, anti-predator behaviour, or nesting success of multiple migrating birds [35, 36, 60]. Differences in hunting behaviour among foxes, driven for example by female reproductive status, may lead to differences in predation risk among and within territories. Finer temporal and spatial scale analyses of predator hunting behaviour may help to better understand fine scale variation in prey distribution and behaviour.

Arctic fox activity budgets may vary on much larger temporal and spatial scales than considered above, due to changing prey availability across time and space. First (seasonal variation), foxes often forage on the sea ice, far away from their territory, to find food during winter [61]. Yet, to our knowledge, no data exists on fox activity budgets in winter. Second (yearly variation), lemming abundance peaks every 3–4 years on Bylot [42] and this influences the intensity of food caching by foxes [31], with likely effects on their activity budgets. Third (spatial variation within Bylot), how much a fox territory overlaps the greater snow goose colony should strongly influence fox activity budget (all foxes studied here lived in the colony), given that spatial heterogeneity of the prey base should induce among-individual differences in hunting behaviour. Fourth (spatial variation across the species distribution), we should expect the activity budget of foxes to strongly vary at the circumpolar scale given the many ecological conditions faced by the species [29]. Better understanding the determinants of fox activity budgets has direct ecological relevance. For example (yearly variations), predation risk on nests of many bird species decreases with lemming abundance on Bylot, likely due to changes in arctic fox behaviour, their shared main predator [62]. Similarly (spatial variation within Bylot), fox predation on artificial nests decreases and shorebird nest abundance increases with distance from the goose colony, where arctic foxes aggregate [34, 60]. Thus, accelerometry data collected on small predators such as arctic foxes over multiple temporal and spatial scales creates new opportunities to shed light on the mechanisms through which predation shapes community structure and function.

### What about acquisition rates and functional responses?

We have shown that quantifying behaviours indicative of foraging (and thus composing a predator’s hunting strategy) offers opportunities to identify predation events. This was, to our knowledge, never achieved before in a small active predator feeding on small prey (50–100 g). Work is still needed, however, to fully estimate acquisition rates of small predators such as the arctic fox. In particular, not all prey items are cached after capture, caching rates can vary with prey availability, and some prey items can be cached and recovered several times. Thus, caching rates do not directly translate into acquisition rates. Future research should seek to differentiate digging events associated to caching of food items such as lemmings, large goose eggs, small passerine eggs, pieces of large mammal carcasses, etc., and differentiate among events of caching, cache recovery, recaching and eating. This will potentially be achieved using modelling techniques that identify microevents [63], or other data sources such as video or audio recorders [3]. At last, such a precise classification would allow the estimation of the predation metrics used to derive functional responses, which are central to predator–prey interactions as they determine links between trophic levels [5, 64].

## Conclusion

We developed a supervised-learning algorithm to classify arctic fox accelerometry data into four main behaviours. This allowed us to assess spatio-temporal variation in fox probability to dig, a behaviour associated with prey caching. In doing so we demonstrated that high precision movement data may be used to study the hunting behaviours of predators of small body size, as long as their foraging behaviours contain diagnostic acceleration patterns. Importantly, the identification of predation events from movement data opens the possibility to estimate predation metrics needed to disentangle the mechanisms structuring predator–prey relationships and trophic networks.

## Supplementary Information

**Additional file 1:** Tables and figures for additional information on the behavioural classification of arctic fox accelerometry data.

**Additional file 2:** Movie clips of running, walking, digging and motionless behaviours in arctic foxes.

## Data Availability

Arctic fox GPS data are available through the Movebank Data Repository at Berteaux, D. 2020, Arctic fox Bylot-GPS tracking, Movebank Study ID 1241071371 (https://www.movebank.org/cms/webapp?gwt_fragment=page=studies,path=study1241071371). The accelerometry and video datasets are available from the corresponding authors on reasonable request.
