# Supplementary material for: Digging into the behaviour of an active hunting predator: arctic fox prey caching events revealed by accelerometry
Source: Mov Ecol. 2021 Nov 27;9:58. doi: 10.1186/s40462-021-00295-1 (PMC8626921; doi:10.1186/s40462-021-00295-1)

Additional file 1

Digging into the behaviour of an active hunting predator: arctic fox prey caching events revealed by accelerometry

Jeanne Clermont*, Sasha Woodward-Gagné, and Dominique Berteaux*

*Correspondence: jeanne_clermb@hotmail.com and dominique_berteaux@uqar.ca

Content:

Additional Tables S1 to S3, pp. 2-5

Additional Figures A-R, pp. 6-14

Additional Tables

Table S1. Sex (M: male, F: female), reproductive status (R: reproductive, NR: non-reproductive), period of accelerometry data collection, data collection duration, and number of 30-sec accelerometry bursts collected for each of 16 arctic foxes studied in 2018 and 2019 on Bylot Island (Nunavut, Canada). The last row provides summary information.

Unique ID	Colour code	Sex	Year	Reproductive status	Data collection period		Data collection duration (days)	Nb of 30-sec bursts
					Start	End		
718	OBBB	M	2018	R	2018-06-29	2018-07-20	22	6681
			2019	R	2019-05-30	2019-07-17	49	12950
746	BVOB	F	2018	NR	2018-07-04	2018-07-29	26	8106
			2019	R	2019-05-25	2019-07-21	58	8674
717	JVOV	F	2018	R	2018-07-01	2018-07-28	28	8398
			2019	R	2019-06-20	2019-06-30	11	3323
737	RVJO	M	2018	R	2018-06-30	2018-07-16	17	5225
			2019	R	2019-06-14	2019-07-17	34	8445
722	OJOO	F	2018	R	2018-06-30	2018-07-19	20	5873
			2019	R	2019-06-17	2019-06-26	10	2856
376	JMVJ	F	2018	R	2018-06-27	2018-07-28	32	9769
			2019	R	2019-06-07	2019-07-14	38	5579
743	ORRR	M	2018	NR	2018-06-29	2018-07-27	29	8811
			2019	R	2019-06-21	2019-07-20	30	9214
747	JBOR	M	2018	NR	2018-07-04	2018-07-17	14	4411
758	BORR	M	2019	NR	2019-06-15	2019-07-12	28	5990
459	OBOB	M	2019	R	2019-06-20	2019-06-30	11	3401
759	RMJJ	F	2019	NR	2019-06-19	2019-07-22	34	8443
755	VJOO	M	2019	R	2019-06-05	2019-07-09	35	8154
757	BBJO	M	2019	NR	2019-06-12	2019-07-18	37	11516
405	RMBR	F	2019	R	2019-06-22	2019-07-20	29	9036
618	VORB	F	2019	R	2019-07-14	2019-07-19	6	1874
623	BOBB	M	2019	R	2019-07-18	2019-07-19	2	547
Summary information		Nb of M: 9<br>Nb of F: 7		Count of R: 17<br>Count of NR: 6			Average: 26	Average: 6838

Table S2. Ethogram used to classify arctic fox behaviour in the video annotation software BORIS. The proportion of time represented by each behaviour within the 2,400-sec training dataset (before it was split into 3-sec sequences) is indicated. Also given is the 4-category behaviour grouping used for accelerometry classification in the web application AcceleRater, and the number of 3-sec sequences obtained for each behaviour category.

Behaviour	Description	Proportion of time in training dataset	Behaviour category	Nb of 3-sec sequences
Running	Form of locomotion used during fast and long-distance relocations	22.78%	Running	146
Walking	Form of locomotion used during slow relocations, usually during short transitions between running and another behaviour	19.13%	Walking	126
Trotting	Form of locomotion intermediate between running and walking	0.24%	(eliminated)	
Egg caching	Digging (usually with tamping and scooping) to cache an egg, fox is stationary and head is down	3.35%	Digging	49
Egg recovering	Digging to recover an egg previously cached by the same or another individual, fox is stationary and head is down	1.11%		
Other digging	Digging to cache or recover an item unseen by the observer, fox is stationary and sometimes eating, head is down	3.13%		
Standing	Body maintained still on four feet, often between running bouts as the fox stops and looks around	6.32%	Motionless	339
Sitting	Sitting with head up	7.80%		
Resting	Lying down, head either up or down	33.30%		
Sitting and scratching	Sitting while scratching with back paw	0.34%	(eliminated)	
Interacting with geese	Approaching a goose nest, often through a sequence of forward and backward movements adjusted to the goose defense behaviour, rapid reactions	1.68%	(eliminated)	
Interacting with another fox	Parent-offspring interactions at a den, rolling and playing	0.82%	(eliminated)	
Total		100.00%		660

Table S3. Average and standard deviation (SD) for 52 statistics (centered and standardized) calculated for 4 behaviour categories across 660 3-sec sequences of the training dataset. X = lateral axis (sway), Y = longitudinal axis (surge), and Z = vertical axis (heave).

Statistic		Behaviour							
		Running		Walking		Digging		Motionless	
		Average	SD	Average	SD	Average	SD	Average	SD
Mean	MeanX	0.01	1.34	-0.75	0.50	0.37	1.33	0.22	0.74
	MeanY	-0.33	0.56	-0.71	0.35	-1.02	1.20	0.55	0.93
	MeanZ	0.40	0.97	0.19	0.48	0.58	0.96	-0.33	1.05
Standard deviation	stdX	1.37	0.92	0.32	0.37	0.06	0.61	-0.72	0.34
	stdY	1.33	0.90	0.37	0.27	0.16	0.46	-0.73	0.43
	stdZ	1.57	0.83	0.00	0.22	-0.07	0.38	-0.67	0.31
Skewness	SkX	-0.26	0.68	-0.04	0.48	-0.43	0.87	0.19	1.21
	SkY	-0.02	1.04	0.00	0.84	-0.05	0.78	0.02	1.07
	SxZ	-0.40	0.44	0.20	0.77	-0.19	0.74	0.12	1.21
Kurtosis	KuX	-0.39	0.22	-0.17	0.35	-0.01	1.19	0.23	1.25
	KuY	0.01	0.50	-0.13	1.20	-0.18	0.51	0.07	1.12
	KuZ	-0.43	0.15	-0.06	0.91	-0.04	0.64	0.21	1.21
Maximum	MaxX	0.90	1.05	0.24	0.80	0.22	0.58	-0.51	0.74
	MaxY	1.10	1.22	0.00	0.63	-0.27	0.76	-0.43	0.61
	MaxZ	1.21	0.68	0.37	0.54	0.23	0.49	-0.69	0.66
Minimum	MinX	-0.94	1.22	-0.58	0.50	-0.02	0.81	0.62	0.48
	MinY	-1.10	0.87	-0.51	0.44	-0.39	0.58	0.72	0.58
	MinZ	-1.36	1.10	0.03	0.27	0.07	0.41	0.56	0.51
Norm*	normX	1.03	0.99	0.54	0.44	0.07	0.76	-0.65	0.62
	normY	0.87	1.05	-0.23	0.31	0.38	0.56	-0.35	0.95
	normZ	0.80	1.20	-0.36	0.36	-0.65	0.70	-0.12	0.90
Cov*	cov(x,y)	-0.47	1.98	0.27	0.25	0.18	0.74	0.08	0.21
	cov(x,z)	1.05	1.72	-0.22	0.19	-0.14	0.58	-0.35	0.05
	cov(y,z)	0.20	2.03	0.11	0.15	-0.05	0.35	-0.12	0.35
r*	r(x,y)	-0.35	1.19	0.42	0.47	-0.17	0.81	0.02	1.02
	r(x,z)	0.54	1.41	0.17	0.50	0.18	0.69	-0.32	0.83
	r(y,z)	-0.04	1.12	0.39	0.44	-0.36	0.71	-0.08	1.09
DBA*	DBA_X	1.44	0.97	0.23	0.31	0.00	0.60	-0.71	0.21
	DBA_Y	1.47	0.87	0.24	0.28	0.14	0.48	-0.74	0.24
	DBA_Z	1.64	0.80	-0.06	0.21	-0.12	0.36	-0.67	0.17
ODBA*	ODBA	1.60	0.67	0.13	0.25	0.00	0.48	-0.74	0.21
Mean difference*	mean-diff_XY	0.26	1.06	0.01	0.47	1.08	1.63	-0.27	0.86
	mean-diff_XZ	-0.16	1.29	-0.62	0.52	0.03	1.29	0.30	0.81
	mean-diff_XZ	-0.49	0.76	-0.74	0.42	-1.22	1.17	0.67	0.69
Standard deviation difference*	std-diff_XY	1.42	0.92	0.23	0.26	0.09	0.38	-0.71	0.38
	std-diff_XZ	1.28	1.11	0.31	0.33	0.02	0.31	-0.67	0.43
	std-diff_YZ	1.39	1.05	0.12	0.22	0.11	0.37	-0.66	0.41
Wave amplitude*	wave amplitude X	1.38	0.86	0.40	0.38	0.07	0.56	-0.75	0.28

	wave amplitude Y	1.42	1.01	0.17	0.29	0.15	0.50	-0.70	0.30
	wave amplitude Z	1.56	0.88	0.02	0.26	-0.03	0.35	-0.67	0.27
Line crossings*	line crossings XY	0.91	0.79	0.74	0.56	0.08	1.11	-0.68	0.61
	line crossings XZ	0.76	0.57	1.17	0.77	0.09	1.00	-0.78	0.29
	line crossings YZ	1.19	0.79	0.24	0.66	0.91	1.17	-0.74	0.16
25 percentile	X 25%	-0.62	1.35	-0.67	0.46	0.28	1.15	0.48	0.58
	Y 25%	-0.70	0.62	-0.78	0.27	-0.89	0.91	0.72	0.75
	Z 25%	-1.11	1.23	0.18	0.33	0.54	0.62	0.33	0.72
50 percentile	X 50%	0.26	1.18	-0.87	0.54	0.38	1.35	0.16	0.79
	Y 50%	-0.29	0.64	-0.73	0.40	-0.98	1.20	0.54	0.92
	Z 50%	0.70	1.22	0.04	0.42	0.43	0.87	-0.38	0.87
75 percentile	X 75%	0.97	0.93	-0.68	0.46	0.42	1.24	-0.23	0.79
	Y 75%	0.24	0.65	-0.48	0.49	-0.98	1.44	0.22	1.04
	Z 75%	1.45	0.55	0.02	0.33	0.22	0.70	-0.67	0.59

* Description of statistics (Resheff et al. 2014[1]):

Norm: The vector norm of the accelerometry sample.

Cov: Covariance between pairs of axes.

r: Pearson's correlation between every two axes.

DBA: Dynamic Body Acceleration by axis. The sum of acceleration values of the axis.

ODBA: Overall Dynamic Body Acceleration. The sum over the axes of the DBA.

Mean difference: The mean difference between every two axes.

Standard deviation difference: The standard deviation of the difference between every two axes.

Wave amplitude: The average difference between consecutive local minima and maxima.

Line crossings: The number of times each two axes cross over each other.

[1] Resheff YS, Rotics S, Harel R, Spiegel O, Nathan R. AcceleRater: a web application for supervised learning of behavioral modes from acceleration measurements. Mov Ecol. 2014;2:27.

Additional Figures

Panels A to R. Boxplots showing variation in the 52 summary statistics (described in Table S3) computed for each 3-sec sequence of the training dataset among the 4 behaviour categories. On all figures: X = lateral axis (sway), Y = longitudinal axis (surge), and Z = vertical axis (heave). Boxplots show first quartile, median, and third quartile. Lower and upper whiskers extend, respectively, to the lowest and highest value within the interquartile range multiplied by 1.5. Black dots represent values outside this range and blue triangles are mean values.

A. Mean

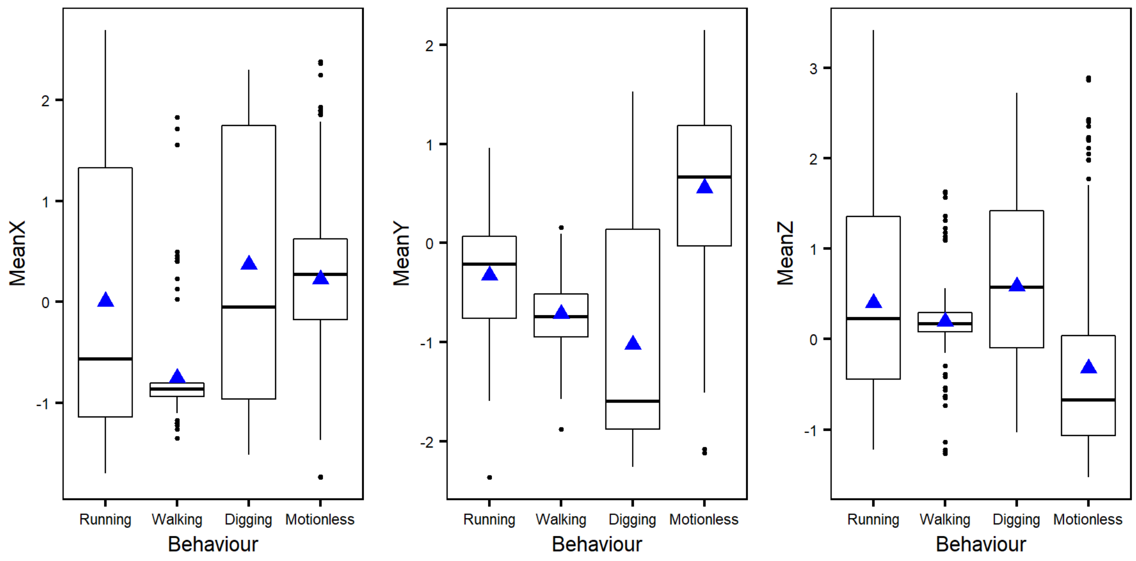

B. Standard deviation

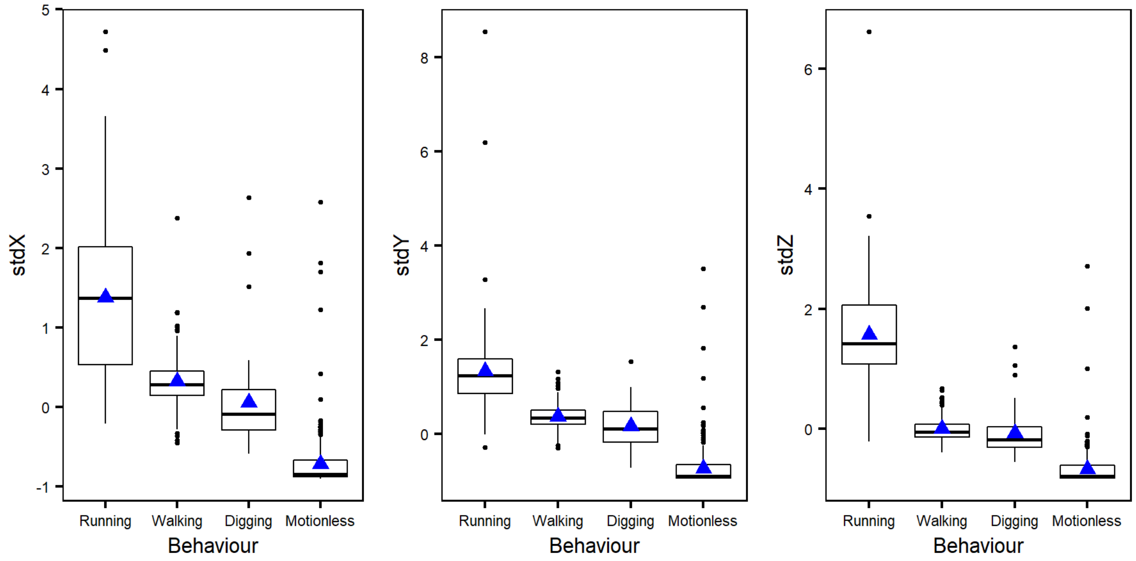

C. Skewness

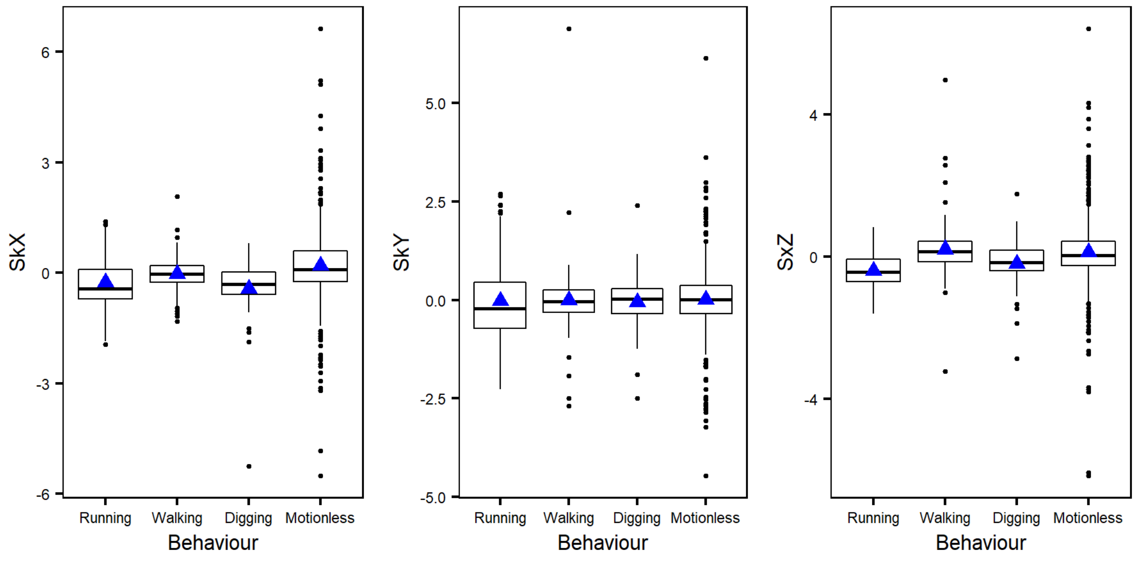

D. Kurtosis

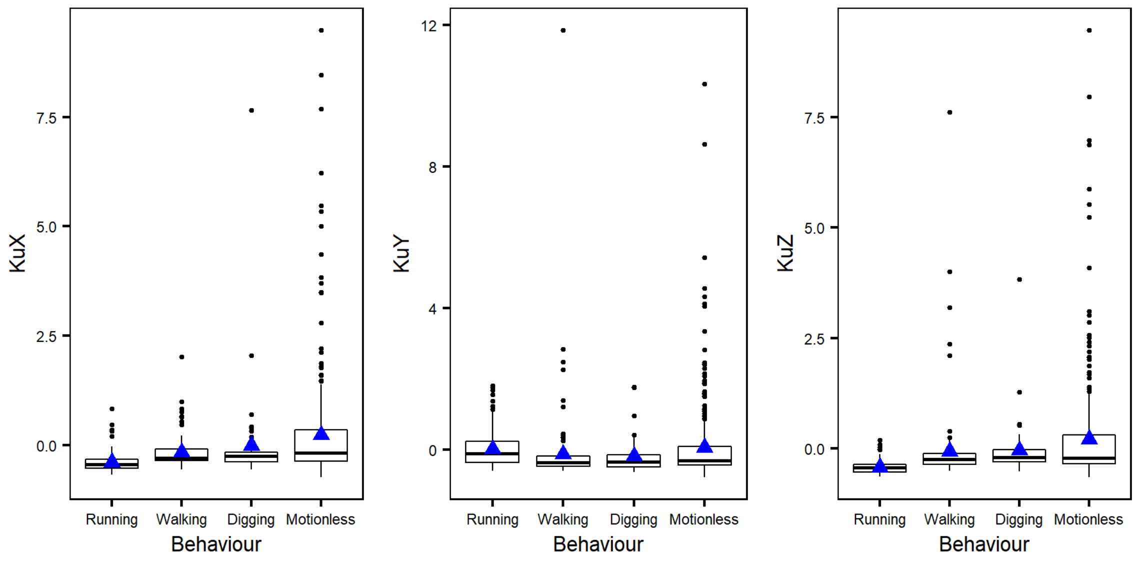

E. Maximum

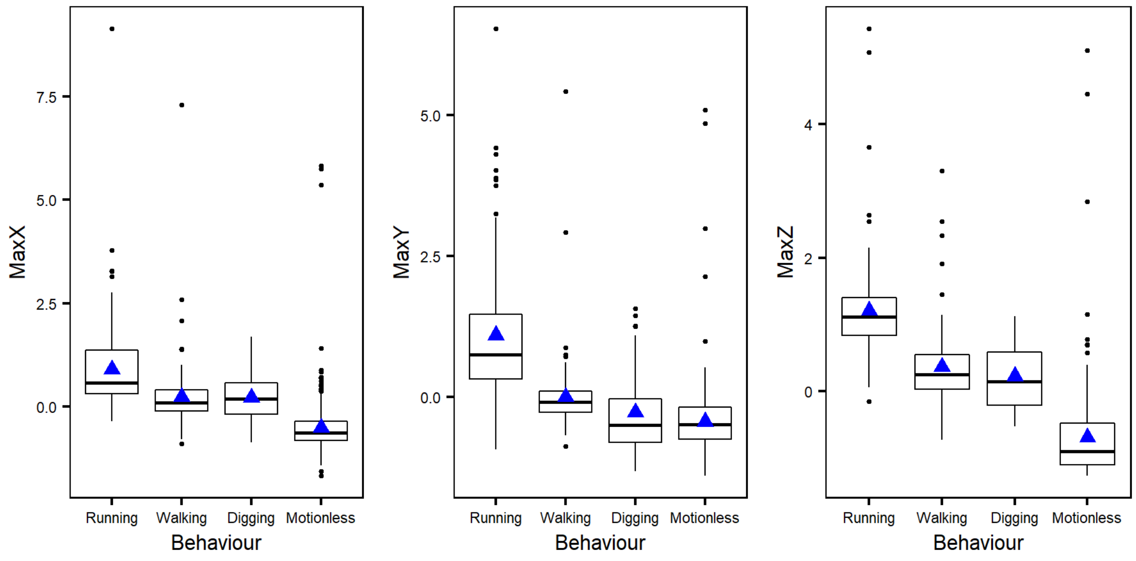

F. Minimum

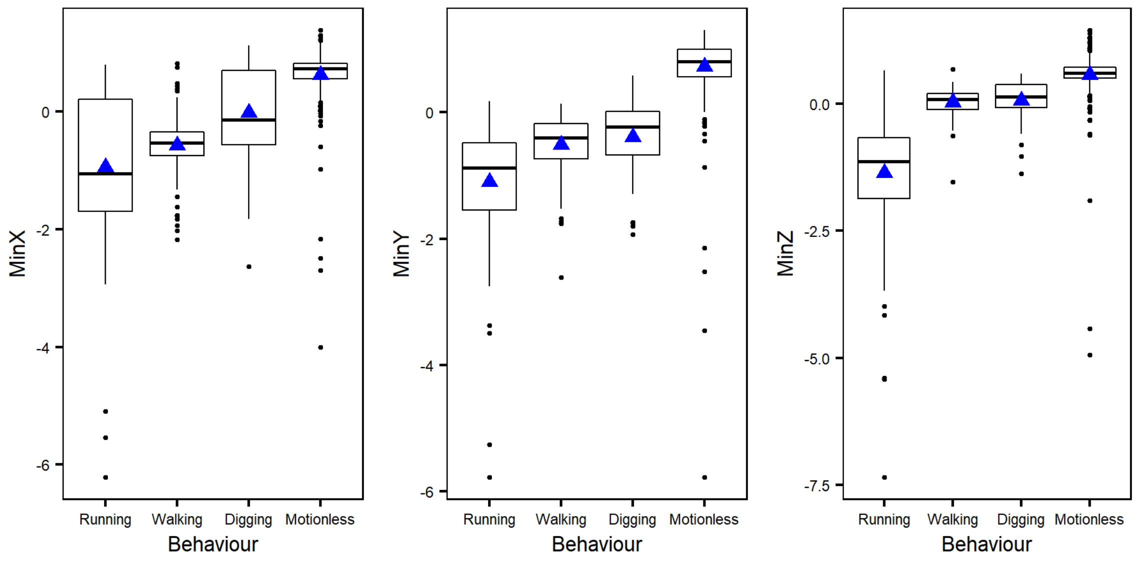

G. Norm

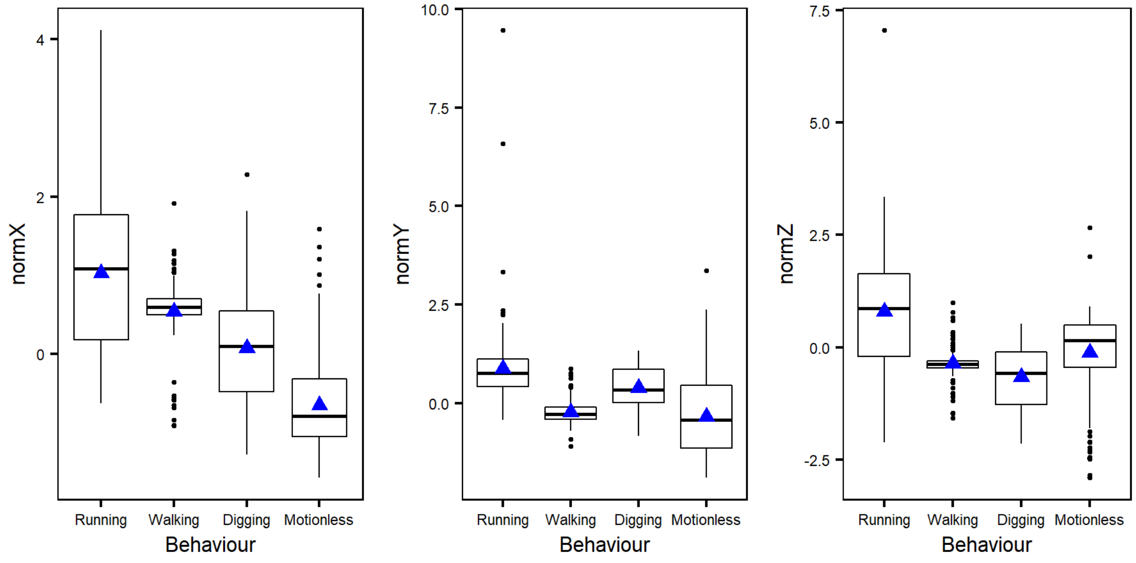
normX
4
2
0
Running
Walking
Digging
Motionless
Behaviour
normY
10.0
7.5
5.0
2.5
0.0
Running
Walking
Digging
Motionless
Behaviour
normZ
7.5
5.0
2.5
0.0
-2.5
Running
Walking
Digging
Motionless
Behaviour

H. Cov

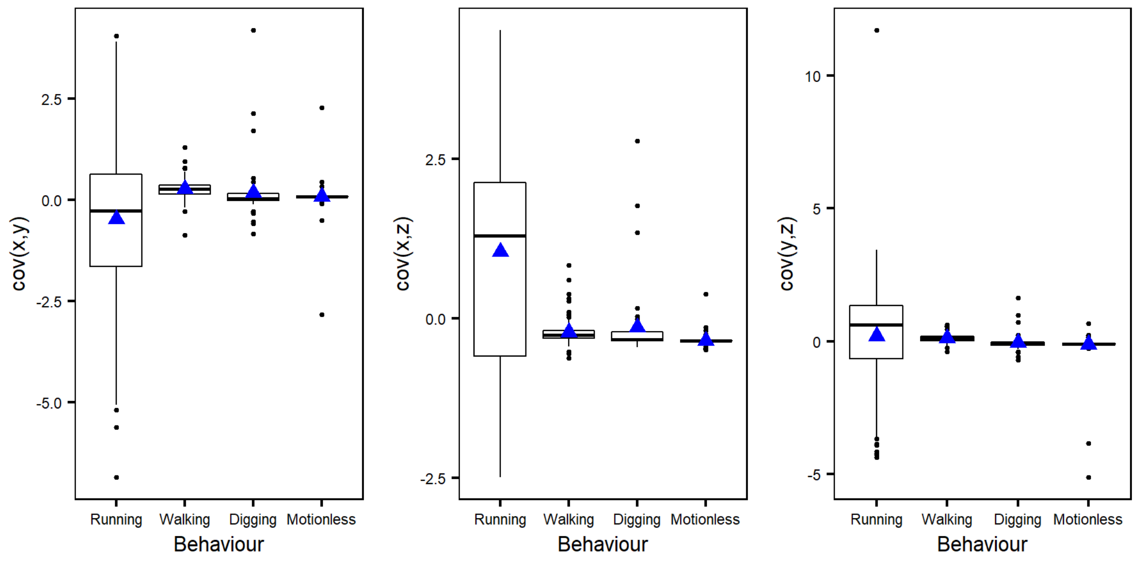
cov(x,y)
2.5
0.0
-2.5
-5.0
Running
Walking
Digging
Motionless
Behaviour
cov(x,z)
2.5
0.0
-2.5
Running
Walking
Digging
Motionless
Behaviour
cov(y,z)
10
5
0
-5
Running
Walking
Digging
Motionless
Behaviour

I. r

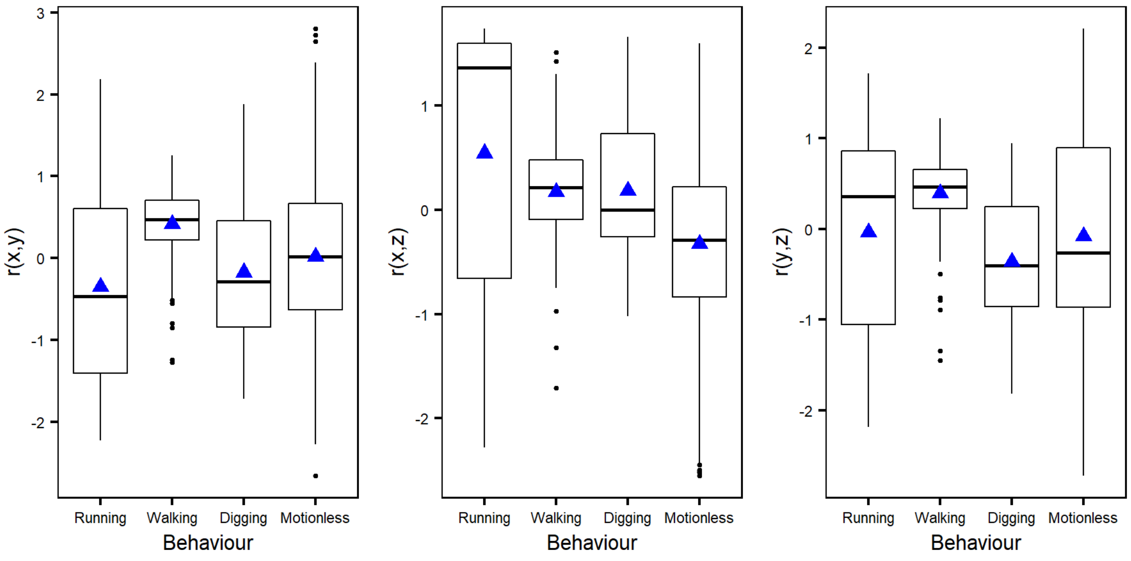

r(x,y)
r(x,z)
r(y,z)
Running
Walking
Digging
Motionless
Behaviour

J. DBA

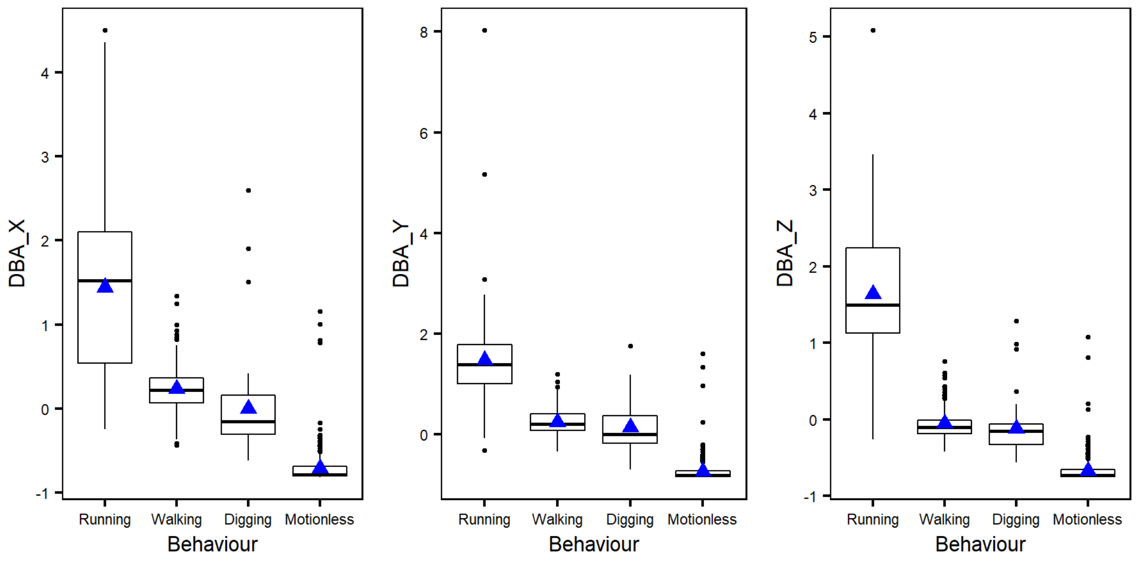

DBA_X
DBA_Y
DBA_Z
Running
Walking
Digging
Motionless
Behaviour

K. ODBA

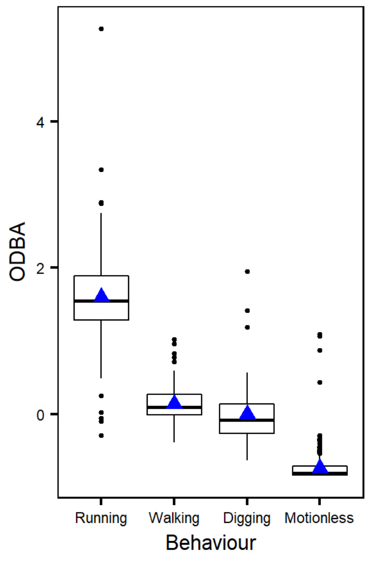

L. Mean difference

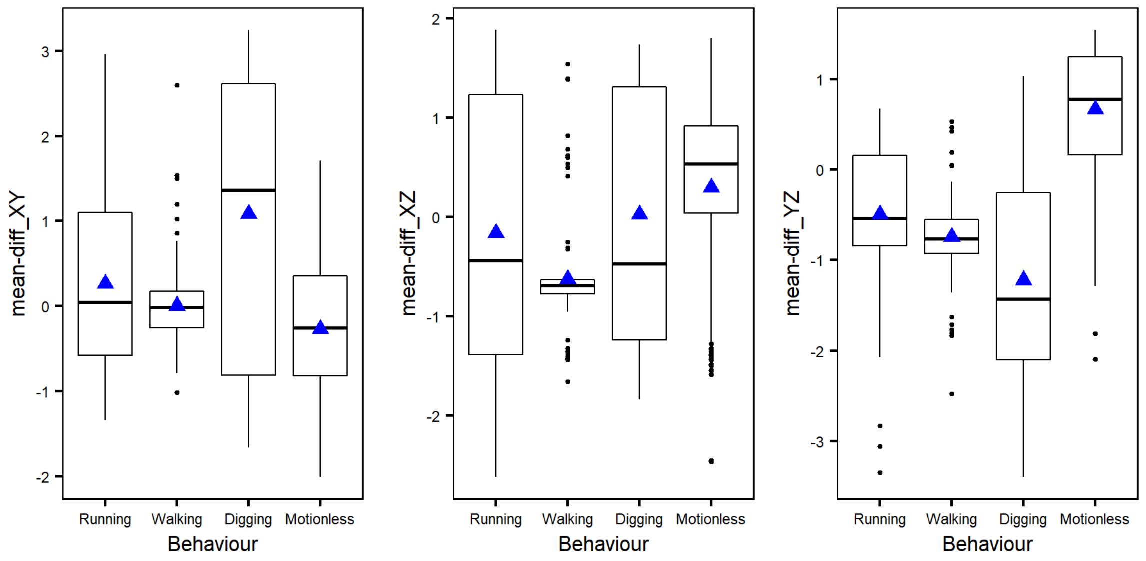

M. Standard deviation difference

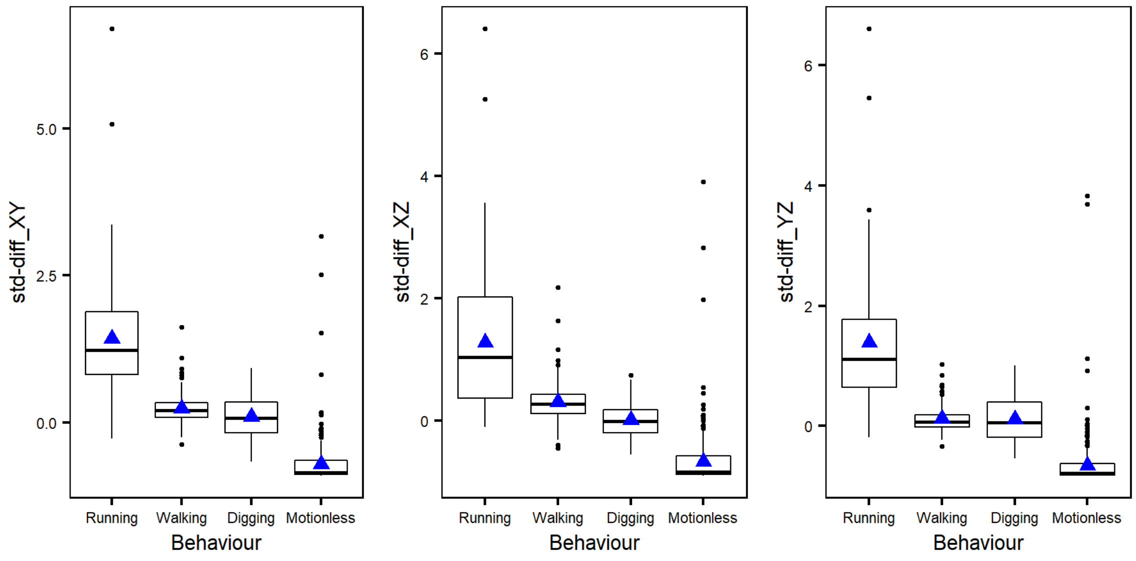

N. Wave amplitude

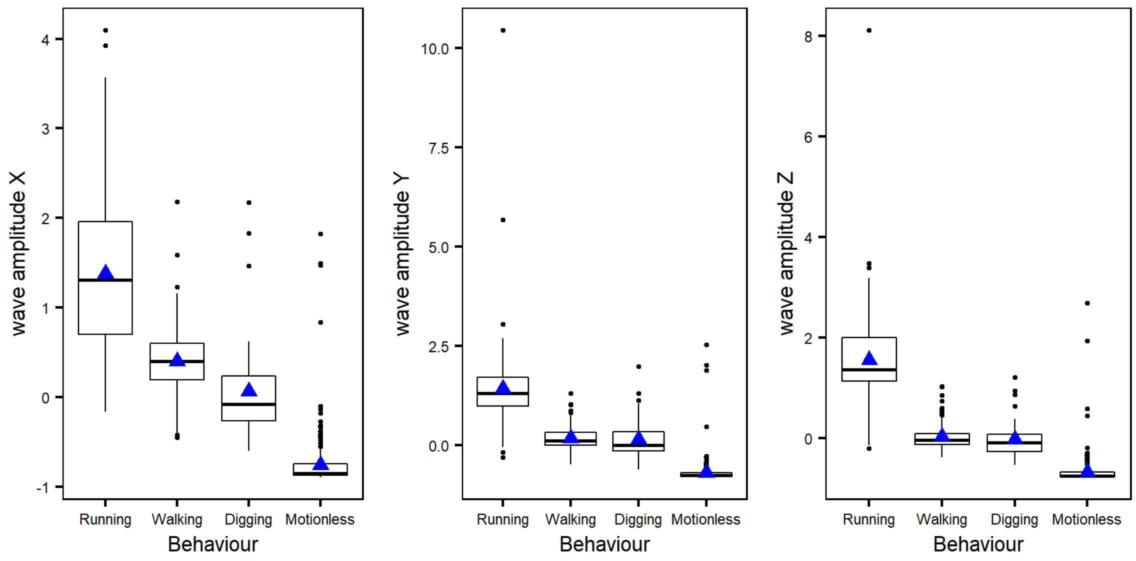

O. Line crossings

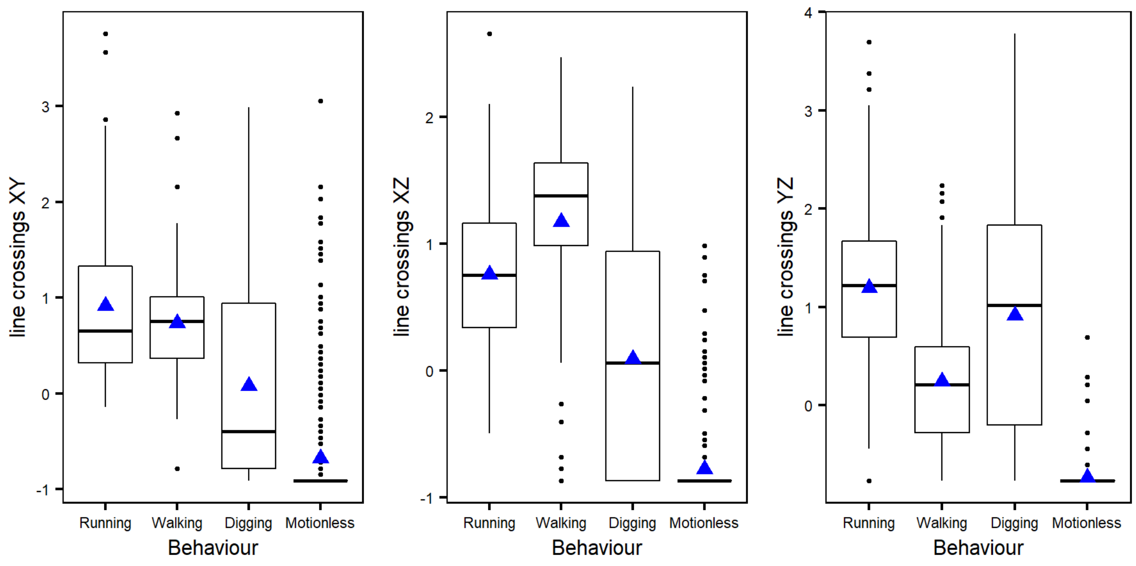

P. 25 percentile

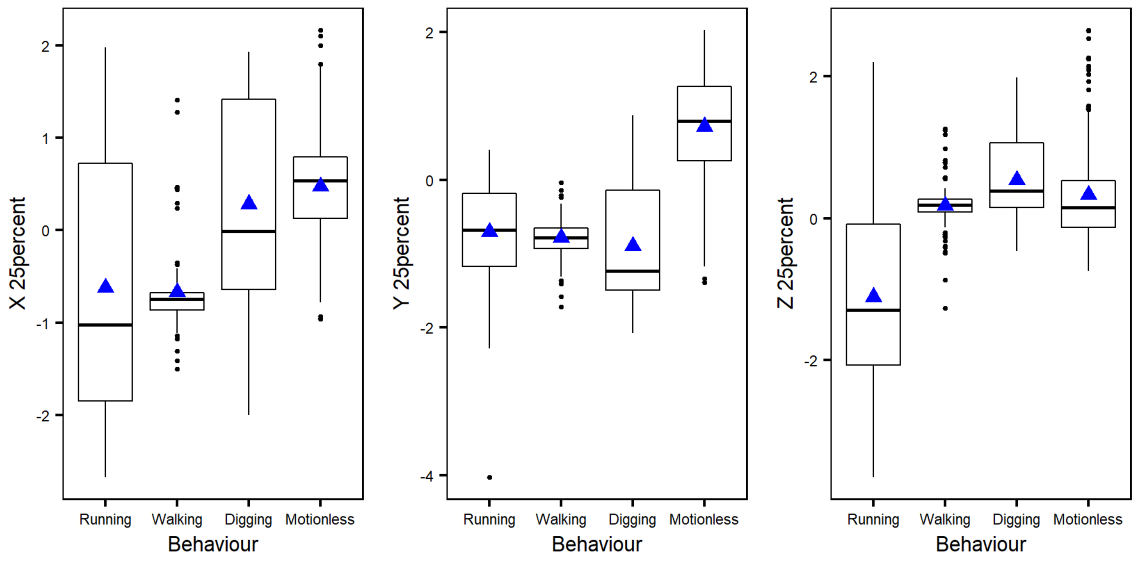

Q. 50 percentile

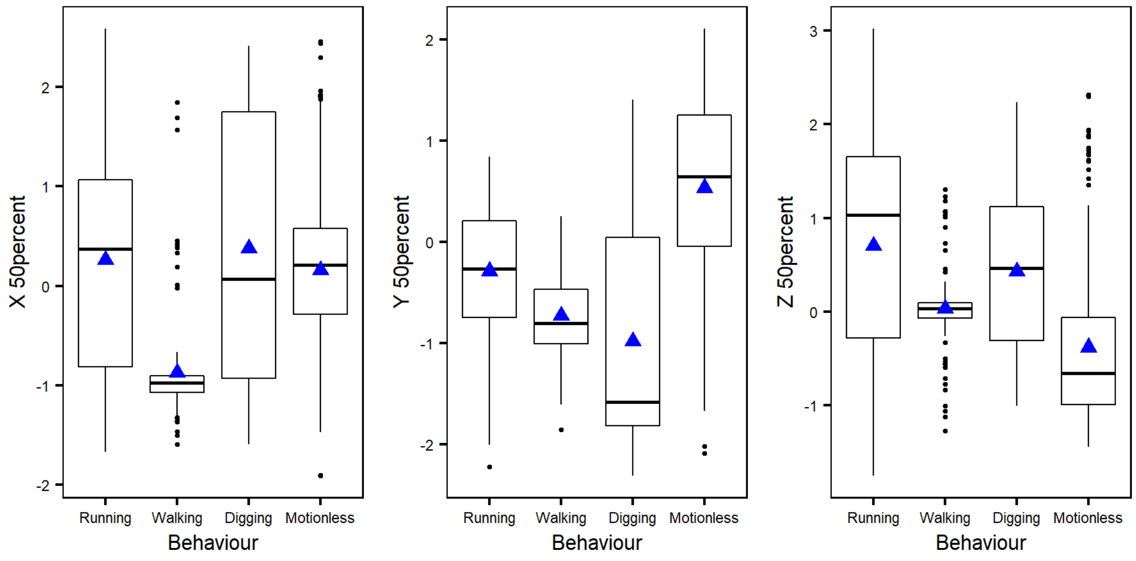

R. 75 percentile

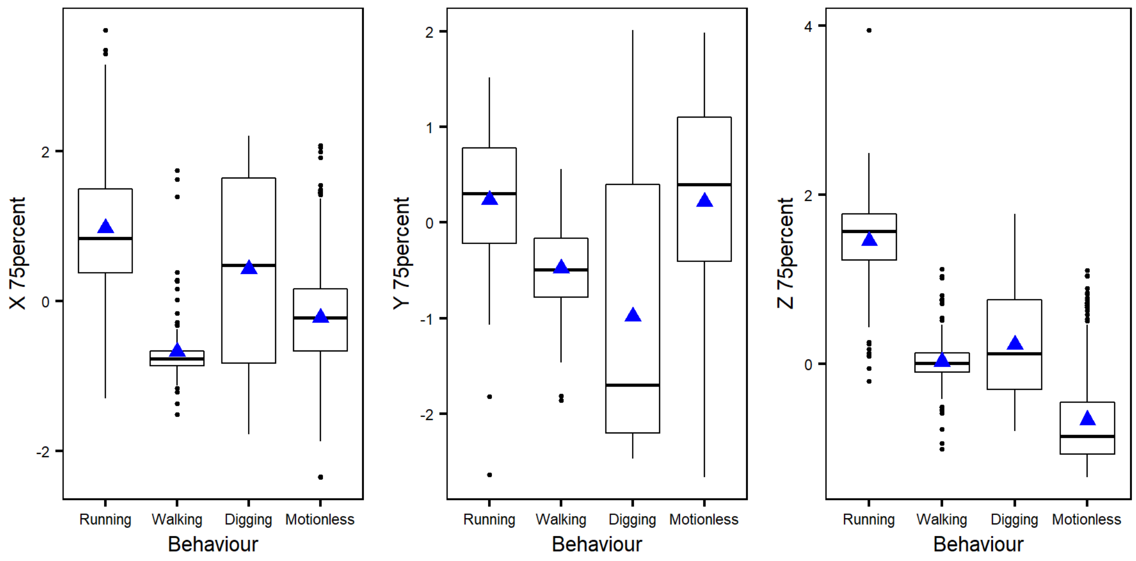

Supplement: Supplementary file 1 — Additional file 1: Tables and figures for additional information on the behavioural classification of arctic fox accelerometry data. [file 40462_2021_295_MOESM1_ESM.pdf]
